# Generation of multidrug resistant human tissues by overexpression of the ABCG2 multidrug transporter in embryonic stem cells

**DOI:** 10.1371/journal.pone.0194925

**Published:** 2018-04-12

**Authors:** Zsuzsa Erdei, Anita Schamberger, György Török, Kornélia Szebényi, György Várady, Tamás I. Orbán, László Homolya, Balázs Sarkadi, Ágota Apáti

**Affiliations:** 1 Institute of Enzymology, Research Centre for Natural Sciences, Hungarian Academy of Sciences, Budapest, Hungary; 2 Institute of Biophysics and Radiation Biology, Semmelweis University, Budapest, Hungary; University of Tampere, FINLAND

## Abstract

The ABCG2 multidrug transporter provides resistance against various endo- and xenobiotics, and protects the stem cells against toxins and stress conditions. We have shown earlier that a GFP-tagged version of ABCG2 is fully functional and may be used to follow the expression, localization and function of this transporter in living cells. In the present work we have overexpressed GFP-ABCG2, driven by a constitutive (CAG) promoter, in HUES9 human embryonic stem cells. Stem cell clones were generated to express the wild-type and a substrate-mutant (R482G) GFP-ABCG2 variant, by using the Sleeping Beauty transposon system. We found that the stable overexpression of these transgenes did not change the pluripotency and growth properties of the stem cells, nor their differentiation capacity to hepatocytes or cardiomyocytes. ABCG2 overexpression provided increased toxin resistance in the stem cells, and protected the derived cardiomyocytes against doxorubicin toxicity. These studies document the potential of a stable ABCG2 expression for engineering toxin-resistant human pluripotent stem cells and selected stem cell derived tissues.

## Introduction

ATP-binding cassette multidrug transporter proteins (MDR-ABC) actively extrude many types of xenobiotics and drugs from the cells, protect our tissues against harmful metabolites and contribute to the resistance of cancer cells against chemotherapy [1]. The most significant human MDR-ABC transporters are ABCG2, ABCB1 and ABCC1, which form a special chemoimmunity network [2]. The ABCG2 protein is a half-transporter, physiologically highly expressed in the liver, intestine, kidney and the tissue barriers, contributing to remove both endo- and xenobiotics, including the toxic compounds of porphyrin metabolism [3–7]. The ABCG2 protein has also been identified in many types of tissue-derived stem cells and in human embryonic stem cell lines (hESC) [8,9]. We have first shown the presence of the ABCG2 transporter in the HUES cell lines and demonstrated the protecting role of this transporter under different stress conditions [10,11]. We have also documented the heterogeneous expression of ABCG2 in hESCs, and found a dynamic balance of ABCG2 expression at the stem cell population level [11].

In the present study, in order to apply a suitably tagged ABCG2 which can be easily followed and visualized in living cells, we used a GFP-ABCG2 fusion protein, shown to be fully functional and correctly processed in various cell types [12]. Moreover, a stable expression of this GFP-ABCG2 in hESCs, by using the Sleeping Beauty transposon system and a CAG promoter, has already been successfully performed by our group [13].

There are several mutant variants of the ABCG2 transporter, which significantly affect the function of this protein. First found in drug-selected cancer cell lines, the R482G-ABCG2 variant was shown to have different substrate recognition and regulation properties [14,15], providing increased resistance against toxic agents, e.g. doxorubicin, than the wild-type protein. In the present study we have generated the GFP fusion constructs of the wild-type and the R482G ABCG2 variants, and characterized their expression, function and trafficking in stem cells. While we did not see an effect of ABCG2 expression on the pluripotency markers, the growth properties, and the morphology of hESCs, they showed an increased resistance against mitoxantrone.

In addition to the characterization of GFP-ABCG2 overexpressing hESCs, we performed detailed differentiation studies, and found a preserved expression and membrane localization of the GFP-ABCG2 variants in the stem cell derived cardiomyocytes and hepatocytes. Since the CAG promoter is especially efficient in cardiac tissues, we examined the potential protective effect of ABCG2 in cardiomyocytes. Indeed, we found that the stem cell derived ABCG2-expressing cardiomyocytes, with preserved beating function, were significantly protected against doxorubicin toxicity.

## Materials and methods

### Maintenance of the undifferentiated human embryonic stem cell lines

Research involving human embryonic stem cells has been approved Hungarian Medical Research Council (6681/2012/HER) and this study was reviewed and approved by a specific ethics committee (Human Reproduction Committee, HRB) of the Hungarian Medical Research Council. The HUES9 human embryonic stem cell line (originally provided by Douglas Melton, Harvard University, in 2005 [16]), its EGFP-expressing derivative (HUES9-EGFP) and GFP-ABCG2 variants expressing HUES9 cell lines were maintained essentially as described before [8]. The cells were cultured on mitomycin-C-treated mouse embryonic fibroblast (MEF) feeder cells in tissue culture 6-well plates (Nunc). The culture medium consisted of 15% Knockout Serum Replacement (Gibco) in Knockout Dulbecco Modified Eagle Medium (Gibco), supplemented with 1 mM Glutamax-I (Gibco), 0.1 mM beta-mercaptoethanol, 1% nonessential amino acids, and 4 ng/mL human fibroblast growth factor (Invitrogen). The media were changed daily and the cells were re-plated every second day onto fresh feeder layers by enzymatic dissociation with 0.025% trypsin-EDTA (Invitrogen). In certain experiments we applied the previously generated and characterized EGFP transgene expressing HUES9 cells, as a control. These EGFP-expressing cells are similar to the parental HUES9 cells in terms of their pluripotency and differentiation properties [13].

### Generation of HUES9 clones expressing GFP-ABCG2 variants

The cDNAs coding for three variants of ABCG2 (wild-type (R482R), R482G, and K86M, see S1 Fig) were transfected together with the SB transposase into hESCs. After 2 days of transfection, we sorted the cells stably expressing GFP-ABCG2, based on the GFP fluorescence by using flow cytometry. After sorting, the cell cultures were maintained in 0.8 μg/ml puromycin containing media and the GFP-ABCG2 expressing clones were picked mechanically. After the establishment of stable GFP-ABCG2 expressing hESC clones, we measured the integrated copy number of the transgenes, based on the IRDR L sequences [17]. We found that the integrated copy numbers in the HUES9 cells were 8 for the wt GFP-ABCG2 R482R, 6 for GFP-ABCG2 R482G and 4 for the GFP-ABCG2 K86M variants.

### Spontaneous differentiation and cardiomyocyte separation

Spontaneous differentiation of the HUES9 and GFP-ABCG2 variants expressing HUES9 cells were performed via embryoid body (EB) formation, as described previously [18]. After 6 days, the EB were placed onto gelatin coated 24 well plates, where they attached to the surface and underwent spontaneous differentiation. Immunostainings of spontaneously differentiating samples were performed at day 6, while real-time PCR measurements were performed at days 6, 12 and 18. For characterization of hESC-derived cardiomyocytes the rhythmically contracting areas were isolated mechanically, and re-plated on gelatin coated 8-well confocal chambers [19]. The immunostaining and doxorubicin treatments were performed at day 24.

### Hepatic differentiation

The differentiation protocol was based on Si-Tayeb et al. 2010 [20] and Cai et al. 2012 [21] protocols, adapted to HUES9 cells. Briefly, to initiate differentiation, HUES9 cells at ~70% confluence in 6-well plates were treated with Accutase (Stem Cell Technologies) and transferred to Matrigel-coated (50 μg/ml) 12 well plates (3x10^5^/cm^2^), and cultured for 24–36 hours to reach ~90% confluence (0–2 days). During this time, mTeSR1 (Stem Cell Technologies) media were applied at 37°C and 5% CO_2_. At the beginning of the differentiation, for endoderm formation, the media were replaced with RPMI supplemented with 2% B27-minus insulin (Gibco), 100 ng/ml Activin A, 10 ng/ml BMP4 and 20 ng/ml FGF2 (all from Peprotech) for two days, with daily media changes at 37°C and 5% CO_2_. In the next 3 days the BMP4 and FGF2 were leaved out from the media, otherwise the same conditions were applied (3–7 days). Hepatic specification was stimulated by RPMI media containing B27-plus insulin (Gibco), 20 ng/ml BMP4 and 10 ng/ml FGF2 (all from Peprotech) for five days at 37°C and 5% CO_2_, and the media were replaced daily (8–12). The next 5 days RPMI supplemented with B27-plus insulin, human recombinant HGF 20 ng/ml (Peprotech) was applied with daily media changes at 37°C and 5% CO_2_ (13–17 days). At the end of the differentiation the medium was changed to Hepatocyte Culture Medium Clonetics HCM (Lonza CC-3198) containing ‘Singlequots’, however the EGF was omitted, and Oncostatin M 20 ng/ml (Peprotech) supplement was added (18–22 days). The media were changed daily for 5 days at 37°C and 5% CO_2_.

### Immunocytochemical staining—confocal microscopy

For immunofluorescence staining, undifferentiated and differentiated HUES9 parental and GFP-ABCG2 expressing HUES9 cells were seeded onto Matrigel coated (50 μg/mL) Imaging dishes (Zellkontakt). For labeling of pluripotency markers and the differentiation markers, we used the following primary antibodies: Oct4 (mouse, monoclonal, 1:50, SantaCruz,), cardiac Troponin-I (mouse monoclonal, 1:300, Sigma), beta-III Tubulin (mouse, monoclonal, 1:2000, RnD Systems), anti-human alpha Fetoprotein (AFP) (mouse, monoclonal, 1:500, Sigma), anti-alpha smooth muscle actin (SMA) (mouse, monoclonal, 1:100, Abcam), HNF4 (rabbit, polyclonal, 1:100, Abcam) and CK18 (mouse, monoclonal, 1:100, Abcam). As a primary antibody against GFP, anti-GFP (rabbit, polyclonal, 1:500, Abcam or chicken, polyclonal, 1:250, AVES) was used. 1 μM DAPI (Thermo Fisher Scientific) was used for nuclear staining. The stained samples were examined by a Zeiss LSM710 confocal laser scanning microscope, using Plan-Apochromat 20x (0.8) air objective (Zeiss) at 405, 488 and 633 nm excitations, respectively.

### Flow cytometry analysis

Cells were suspended in PBS, containing 0.5% bovine serum albumin, and labeled with a combination of monoclonal antibodies (mAbs). For all samples an anti-mouse Sca-1 (Ly-6A/E) PE or FITC (BD Pharmingen) antibody was used, for gating out the positively labeled mouse feeder cells. For specific labeling of HUES9 cells we used the directly labeled anti-human SSEA4-APC antibody (R&D System). Indirect staining of ABCG2 was performed by using the 5D3 monoclonal antibody, applied in the presence of 1 μM Ko143, a specific inhibitor of ABCG2, which maximizes 5D3 binding [15]. The secondary antibody used was R-phycoerythrin conjugated goat anti-mouse IgG2b, (Invitrogen). Control staining with appropriate isotype-matched control mAbs was included.

Cells were incubated for 30 min at 37°C with the antibodies, and dead cells were gated out on the basis of 7AAD (Sigma) staining. Samples were analyzed by a FACSCanto flow cytometer (Beckton-Dickinson) equipped with a 488 nm argon laser and a 635 nm red diode laser, with CellQuest acquisition software (BDIS).

### Real-time PCR analysis

Total RNA was isolated from cells by the use of TRIzol reagent (Invitrogen), in accordance with the manufacturer’s instructions. cDNA samples were prepared from 0.5 μg total RNA by using the Promega reverse transcription system kit, as specified by the manufacturer. For real-time quantitative PCR, pre-developed Taq-Man assays were purchased from Applied Biosystems, for detecting total ABCG2 (Hs01053790_m1), NANOG (HS02387400_g1), Oct4 (POU5F1- Hs00999632_g1), AFP (Hs00173490_m1), T-Brachyury (Hs00610080_m1), Pax6 (Hs00240871_m1) and PRLP0 ribosomal protein (Hs99999902_m1) mRNA, the latter used as the internal control for quantification. We measured the mRNA levels of the Oct4 (POU5F1), AFP (Hs00173490_m1), ALB (Hs00910225_m1), ABCB11 (Hs00184824_m1), and HNF4 (Hs00604435_m1) during hepatocyte differentiation. Relative mRNA levels were calculated by using the 2-DDCt method. TaqMan assays were run and analyzed by using the StepOneTM real-time PCR system (Applied Biosystems), in accordance with the manufacturer’s instructions.

### Dye cycle violet (DCV) transport measurements

We measured the transport function of the GFP-ABCG2 protein variants in HUES9 cells by measuring Dye cycle violet (DCV) fluorescent dye uptake. In these experiments the cells were incubated with 100 nM DCV for 16 hours, in the presence or absence of the ABCG2 specific inhibitor Ko143. The DCV fluorescence signal, representing DNA-bound DCV, was followed in the cells by flow cytometry. The transport activity of ABCG2 was characterized by the “transport activity factor” (F), calculated as follows: (F100-F0)/F100 x100, where F0 is the fluorescence (median values) of DCV-treated cells in the absence of an inhibitor; F100 is the fluorescence (median values) in the presence of the inhibitor Ko143 [22].

### Mitoxantrone (MX) toxicity

HUES9 or HUES9 cells expressing the GFP-ABCG2 variants were seeded onto mitomycin-treated MEF cells. 24 hours later the cells were exposed to 5–200 nM Mitoxantrone (MX). The culture media were changed on the second day. On the third day cells were collected from the MEF cells by trypsin treatment, and analyzed by flow cytometry. The survival of parental and GFP-ABCG2 expressing HUES9 cells was measured based on propidium-iodide accumulation. For all samples an anti-mouse Sca-1 (Ly-6A/E)-PE (Beckton-Dickinson) antibody was used for gating out the positively labeled mouse feeder cells. The ratio of the dead and living cells was calculated on the basis of propidium-iodide accumulation.

### Doxorubicin treatment

The beating cardiomyocyte colonies derived from HUES9 parental and GFP-ABCG2–R482G expressing HUES9 cells were isolated mechanically and re-plated onto gelatin coated 8 well confocal chambers. After 2 days post-plating, the cells were incubated with 3 μM doxorubicin (DOX) for 16 hours. DOX accumulation in the cells was examined by a Zeiss LSM710 confocal laser scanning microscope, using a Plan-Apochromat 20x (0.8) air objective (Zeiss) at 458 nm excitation and 535–595 nm emission wavelength. The survival of parental and GFP-ABCG2 expressing HUES9 derived cardiomyocytes was measured based on propidium-iodide accumulation at 543 nm excitations and 662–797 nm emission wavelength and Hoechst33342 dye accumulation at 405 nm excitations and 410–538 nm emission wavelength.

## Results

### Generation and characterization of hES cell clones expressing GFP-ABCG2 variants

In order to explore the effects of ABCG2 overexpression in hES cells, we have generated HUES9 cells expressing a GFP-tagged version of ABCG2 protein and its variants, driven by a constitutively active CAG promoter. For gene delivery and stable expression of the wild type and the R482G-ABCG2 proteins we used the Sleeping Beauty transposon system (see Methods and S1 Fig), as we have shown earlier that a GFP-tagged version of ABCG2 is fully functional and can be efficiently used to follow the expression, trafficking and function of this transporter in living cells [12]. As a control, we have also generated stem cells expressing a non-functional K86M-ABCG2. hES clones containing 4–8 copies of the transgene and stably expressing the GFP-ABCG2 variants were selected by puromycin, sorted by flow cytometry, and finally picked mechanically (see Methods).

**Fig 1:**
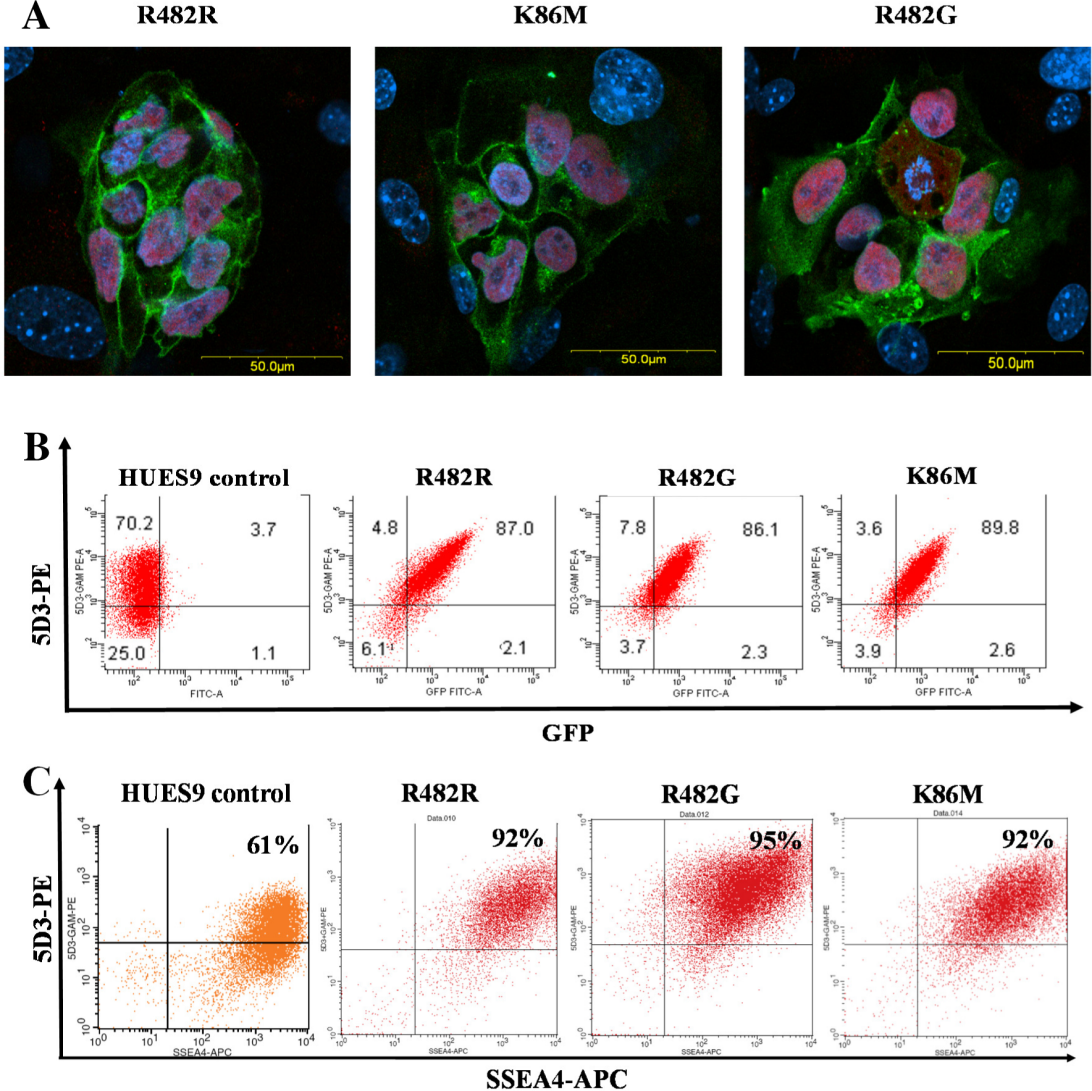
Expression of the GFP-tagged ABCG2 variants in HUES9 cells. (A) Confocal images for HUES9 cell clumps expressing the GFP-ABCG2 variants (green), HUES9 cells are cultured on mouse embryonic fibroblasts. Immunostaining for Oct4 (red), cell nuclei are stained with DAPI (blue). (B) Flow cytometry analysis of HUES9 cells expressing the GFP-ABCG2 variants. The cells were labeled by a monoclonal anti-ABCG2 antibody (5D3), and a phycoerythrin (PE) coupled anti-mouse IgG2b. The green fluorescence of GFP-ABCG2 is plotted against 5D3-PE red fluorescence. Control HUES9 cells, HUES9 cells expressing GFP-ABCG2-R482R, GFP-ABCG2-K86M, and GFP-ABCG2-R482G variants are shown. (C) Flow cytometry analysis of the pluripotency marker SSEA4 in parental HUES9 cells and the GFP-ABCG2-expressing variants. The cells were labeled by a monoclonal anti-ABCG2 antibody (5D3) visualized by a phycoerythrin (PE)-conjugated anti-mouse IgG2b, and by anti-SSEA4, conjugated to APC (green). HUES9 cells expressing GFP-ABCG2-R482R, GFP-ABCG2-K86M, and GFP-ABCG2-R482G variants are shown.

Confocal microscopy measurements (Fig 1A) of these clones showed that the GFP-ABCG2 protein was relatively uniformly expressed in the HUES9 cells, and why intracellular localization was also observable, all three ABCG2 variants localized partially in the plasma membrane of the living hES cells (Fig 1A). Flow cytometry measurements, by using the cell surface reactive anti-ABCG2 antibody, 5D3, also confirmed a relatively high plasma membrane localization of the GFP-ABCG2 in the transgenic clones (Fig 1B). From the numerous clones stably expressing the GFP-ABCG2 protein, we have selected three relevant cell lines for further differentiation studies.

In these experiments we have also examined how the overexpression of GFP-ABCG2 changes the pluripotency and the related marker expression of the hES cells. As documented in Fig 1A, the expression of Oct-4 transcription factor protein was present in the nuclei of all GFP-ABCG2 expressing hES clones. A similar, high level expression at the mRNA level was observed for another key pluripotency marker, Nanog, in the GFP-ABCG2 expressing clones as compared to the parental cell line (see S2 Fig). For further verifying the pluripotent state of the GFP-ABCG2-hES cells, we also measured the expression of the cell surface marker SSEA-4 in the hESCs by using confocal microscopy (data not shown) and flow-cytometry (Fig 1C). As shown in Fig 1C, the cell surface expression of SSEA4, measured by flow cytometry, was uniformly high (over 92%) in the parental and in all HUES cell clones expressing the GFP-ABCG2 variants. In these experiments we have also compared the growth properties of the GFP-ABCG2-hESCs and parental hESCs cell lines. According the similar passaging times and a cell proliferation assay with PrestoBlue, we found no difference between the growth properties of the GFP-ABCG2 expressing and the parental HUES9 cells (data not shown). We also examined the expression of the other main ABC transporters, ABCB1 and ABCC1 by RT-qPCR and found that the overexpression of the ABCG2 variants did not alter the mRNA expression of ABCB1 and ABCC1 in the pluripotent stem cells (S3 Fig). All these results suggest that the overexpression of GFP-ABCG2 in these experiments does not change the pluripotent state and growth properties of the hESCs.

### Spontaneous and directed differentiation of the GFP-ABCG2 expressing HUES9 clones

**Fig 2:**
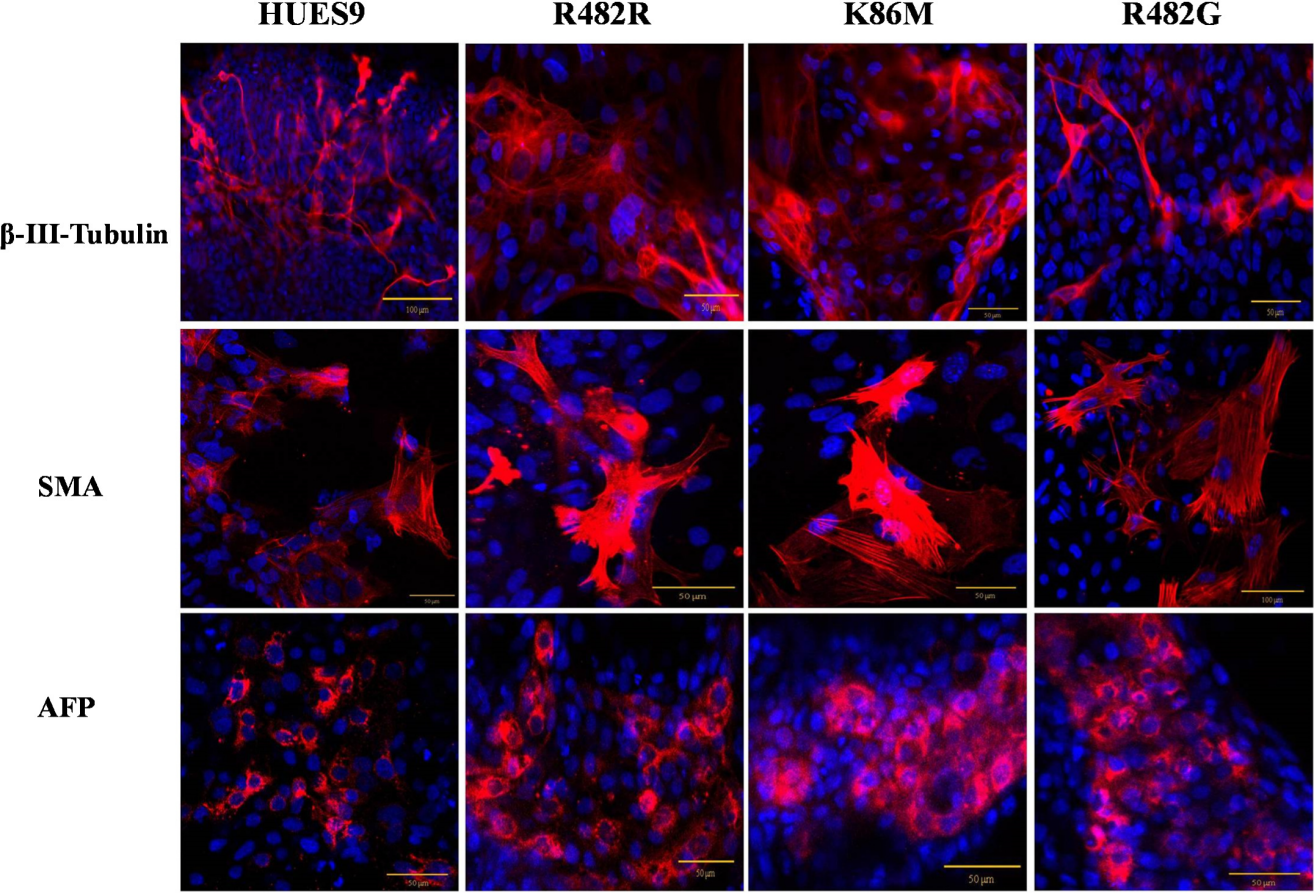
Spontaneous differentiation capacity of the parental HUES9 cells and HUES9 cells expressing GFP-ABCG2 variants—confocal microscopy analysis. Both the parental and the GFP-ABCG2 expressing HUES9 cells express the marker proteins β-III-Tubulin, SMA and AFP (red). Nuclei are stained by DAPI (blue).

For investigating the spontaneous differentiation capacity of the GFP-ABCG2-hES cells into cell types of the three germ layers, we differentiated the GFP-ABCG2-HUES9 cells via the embryoid-body (EB) system (see Methods and [18]). As shown in Fig 2, after 6 days of EB formation and a further 3 days differentiation on an adherent surface, we detected the expression of the key markers specific for the three germ layers, that is β-III-Tubulin as an ectoderm marker, alpha-fetoprotein (AFP) as an endoderm marker, and smooth muscle actin (SMA) as a mesodermal marker protein. We found that these marker proteins were expressed in the differentiated GFP-ABCG2-hESCs similarly to those in the parental hESCs (see Fig 2).

In order to follow the effects of the GFP-ABCG2 transgene expression on spontaneous hESC differentiation, we have also measured a set of mRNA expression levels, characteristic either for the pluripotent state (Oct-4 and NANOG), for ectodermal differentiation (Pax6), endoderm formation (AFP), or mesoderm formation (Brachyury), by real-time PCR (S2 Fig). Again, we found no difference in the expression patterns of these markers between the transgenic and the parental clones. These results collectively indicate that there are no significant differences between spontaneous differentiation capacity of the GFP-ABCG2-hES and that of the parental hESCs.

**Fig 3:**
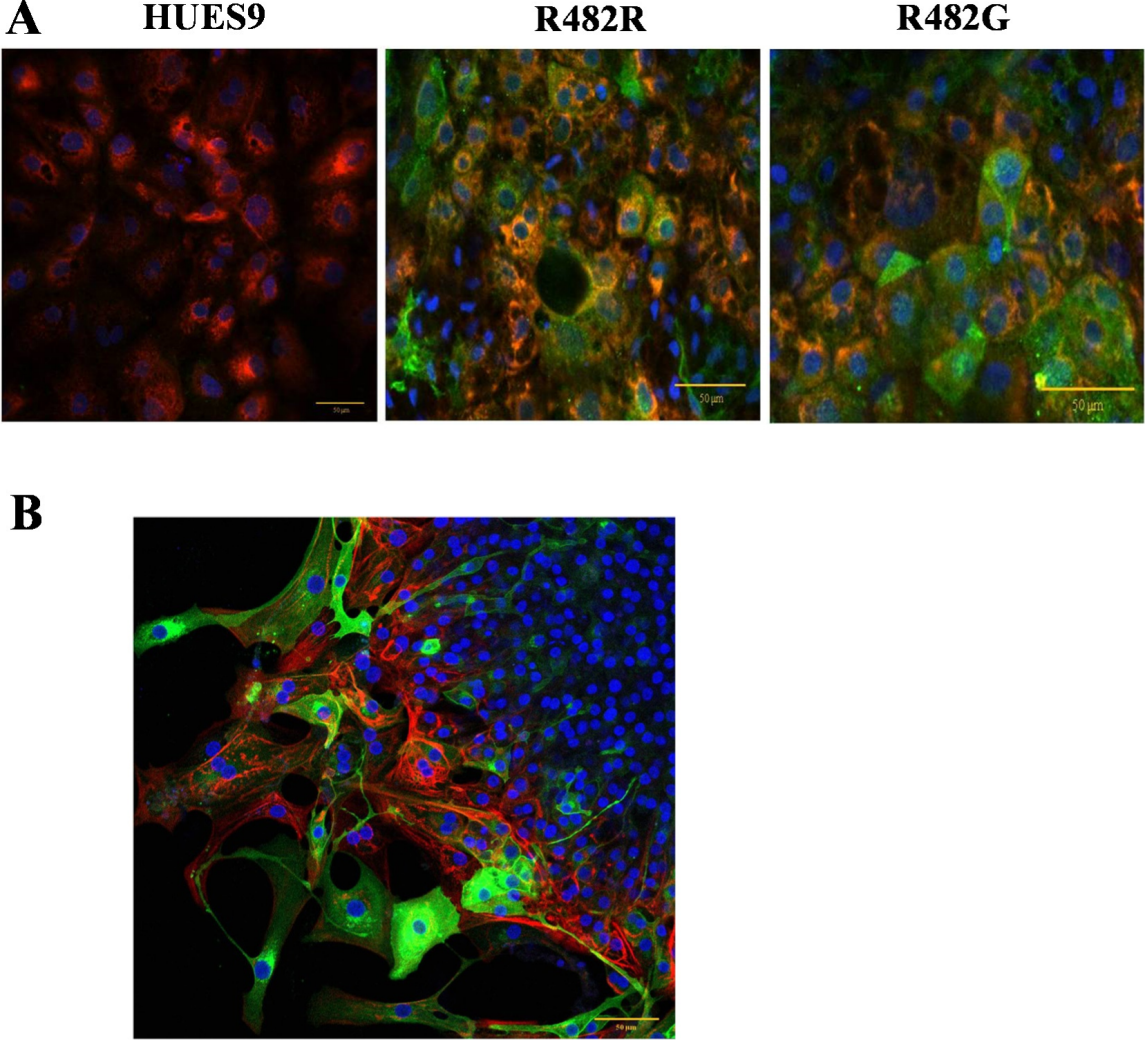
**Directed differentiation of HUES9 cells expressing GFP-ABCG2 into hepatocytes (A) and cardiomyocytes (B).** Confocal immunostainings (A) Co-immunostaining of AFP and GFP-ABCG2 in hepatocytes differentiated from HUES9 cells. Anti-GFP: green, AFP: red, nuclei: blue (B) Co-immunostaining of cardiac troponin I (red) and GFP-ABCG2 (green) in cardiomyocytes differentiated from HUES9 cells. Cell nuclei are stained with DAPI (blue).

When examining the differentiation capacity of the GFP-ABCG2-hESC clones in the direction of hepatocytes, we used protocols for hepatocyte differentiation according to references [20] and [21], adapted to HUES9 cells (see Methods). When measuring the mRNA expression of the ABCB11 transporter, and several other hepatocyte specific genes (HNF4, AFP, ALB) in the undifferentiated cells and in the differentiated hepatocytes by real-time PCR, we found no significant differences in the expression patterns of these markers in the GFP-ABCG2 expressing versus the parental hESCs (S4 Fig). Moreover, when performing antibody–based immunocytochemistry studies in the differentiated hepatocytes, we found a co-expression of AFP, HNF4, CK18 and GFP-ABCG2 (Fig 3A and S5 Fig). These results suggest that the overexpression of GFP-ABCG2 does not affect the directed differentiation capacity of the hESCs into hepatocytes.

Since protein expression driven by the CAG promoter is expected to result in especially high levels in cardiac tissues [13,23], we focused on studying the ABCG2 expression in cardiomyocytes. In order to examine the effects of transgene expression on cardiomyocyte differentiation, we isolated beating cardiomyocyte colonies derived from parental or GFP-ABCG2 expressing hESCs mechanically. In all cell clones expressing the GFP-ABCG2 variants we could obtain beating cardiomyocyte colonies in similar numbers. As shown in confocal microscopy measurements, presented in Fig 3B, the cardiomyocytes derived from the GFP-ABCG2-R482G hESCs co-express the cTnI and the GFP-ABCG2 protein. All these data indicate that stable expression of GFP-ABCG2 does not significantly affect cardiomyocyte differentiation from HUES9 cells, and in these cells pacemaker and contractile activities are preserved. The videos presented in the S Information confirm the similar beating activity of the cardiomyocytes derived from the parental or GFP-ABCG2 expressing hESCs.

### GFP-ABCG2 functional studies in hES cells

After the investigation of the basic characteristic of the GFP-ABCG2-hESCs we analyzed the function of the GFP-ABCG2 in these pluripotent cells. In the following measurements we focused on functional variants i.e. GFP-ABCG2wt and GFP-ABCG2 R482G. As documented above, confocal microscopy measurements showed that a large fraction of the GFP-ABCG2 is localized in the plasma membrane, and in the following experiments we assessed the transport function of the plasma membrane GFP-ABCG2 by measuring the extrusion of a known substrate of ABCG2, DyeCycleViolet (DCV), a fluorescent nuclear stain [24–26].

**Fig 4:**
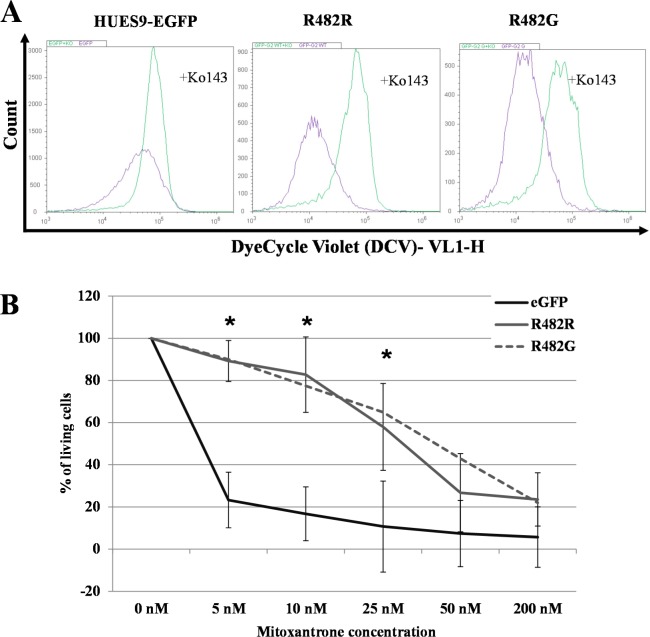
**Functional analysis of ABCG2 in EGFP-HUES9 (control) and GFP-ABCG2 expressing cells** (A) Examination of the transport function of the GFP-ABCG2 protein variants in HUES9 cells by measuring DCV uptake. Cellular DCV fluorescence was measured in cells expressing the R482R (wild-type) and R482G (substrate mutant) variants of GFP-ABCG2. The purple histogram shows the signal of the DCV in the control cells and the green histogram shows the signal of DCV in the Ko143-treated cells. (B) Analysis of mitoxantrone cytotoxicity in EGFP-HUES9 (control) cells and in HUES9 cells expressing GFP-ABCG2 variants. Survival of parental and GFP-ABCG2 expressing HUES9 cells was measured in the presence of 5–200 nM Mitoxantrone (MX) by flow- cytometry. The ratio of the dead and living cells was calculated on the basis of propidium-iodide accumulation and was normalized to untreated cells. Values represent the means±S.D. of 3 independent experiments. Significant differences (Student’s t-test, P<0.01) in the survival of parental and ABCG2-variants expressing clones are indicated by asterisks. The exact mean±S.D. and p values are presented in S1 Table.

In the experiments shown in Fig 4A, hESCs were incubated with 100 nM DCV for 16 hours, in the presence or absence of the ABCG2-specific inhibitor, Ko143. The DCV fluorescent signal, representing DNA-bound DCV, was followed in the cells by flow-cytometry analysis. According to these results, hESCs expressing the wt GFP-ABCG2 or the GFP-ABCG2-R482G have significantly higher DCV extrusion capacity than the parental hESCs (transport activity factors were F_parental_ = 56.65±10.54; F_R482R_ = 82.22±1.86 p = 0.014; F_R482G_ = 78.60±2.26 p = 0.024).

In Fig 4B we document that functional ABCG2 overexpression and higher dye extrusion capacity in the HUES9 cells results in a protective effect against mitoxantrone (MX). In these experiments we incubated the GFP-ABCG2-hESCs and the parental hES cells in media containing 5–200 nM MX respectively, and the survival of the cells was measured by flow-cytometry. These results show that while parental hES cells die at low concentration of MX, the hESCs expressing the wild-type or the GFP-ABCG2-R482G proteins, tolerate MX in much higher concentrations.

### Cardiomyocytes derived from the hESCs expressing the GFP-ABCG2-R482G protein show doxorubicin resistance

As shown above, we successfully isolated beating cardiomyocytes (see also S videos), expressing the cardiomyocyte specific Troponin-I (cTnI), derived from hESCs clones expressing GFP-ABCG2-R482G. In these cardiomyocytes we examined a potential protective effect of the overexpressed GFP-ABCG2-R482G against the cardiotoxic anticancer agent, doxorubicin (DOX). The R482G ABCG2 variant was selected for these experiments because its higher reported transport activity regarding DOX (see [14]).

**Fig 5:**
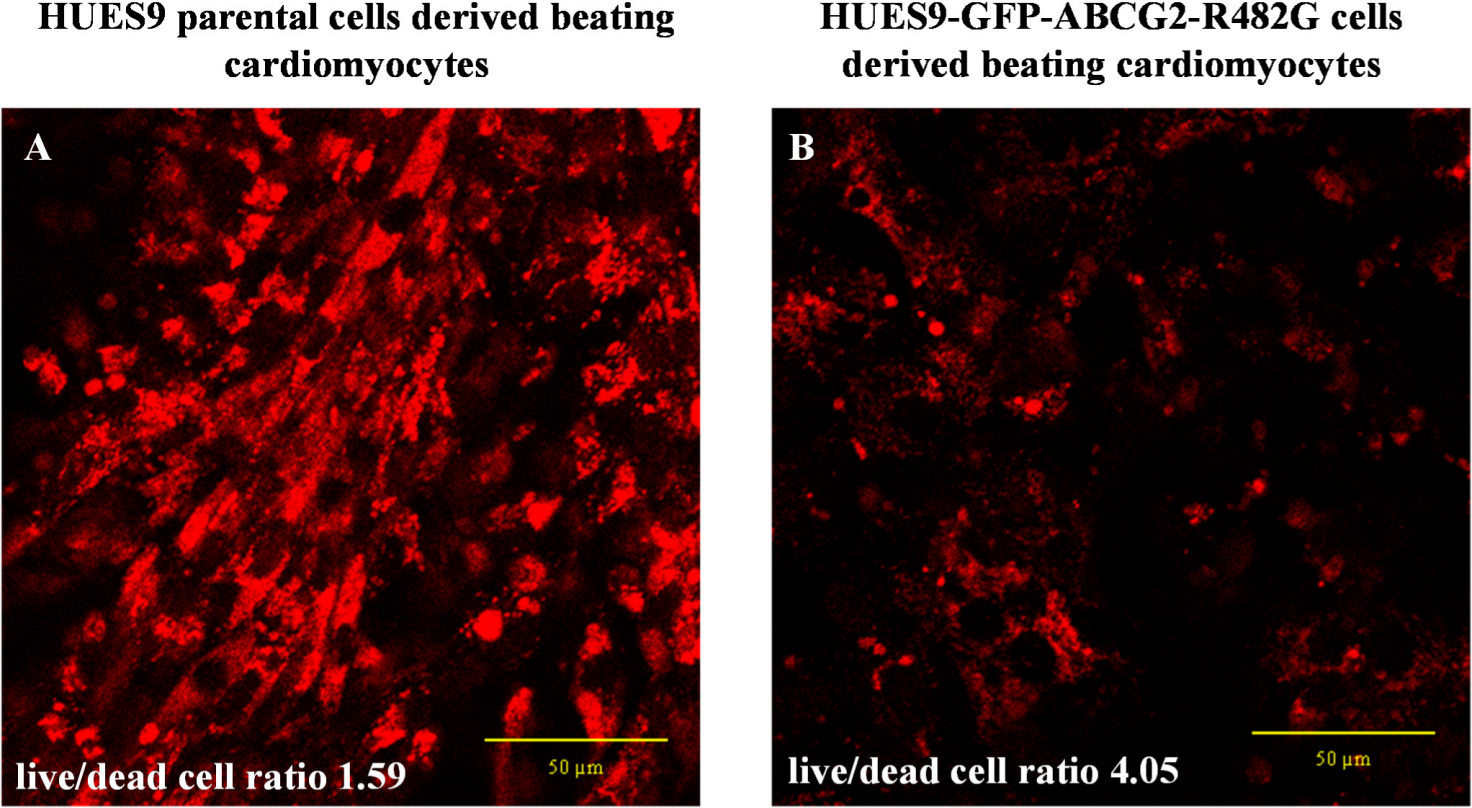
Beating cardiomyocyte colonies derived from HUES9 cells, incubated with doxorubicin (DOX, 3 μM) for 16 hours. DOX accumulation in the cells was assessed by confocal microscopy (see Methods). (A) Cardiomyocytes differentiated from parental HUES9 cells, (B) cardiomyocytes differentiated from HUES9 cells expressing GFP-ABCG2-R482G.

As shown in Fig 5, hESC-derived cardiomyocytes overexpressing the GFP-ABCG2-R482G protein were significantly protected against doxorubicin (DOX) accumulation. The cellular fluorescence of DOX was significantly lower in hESCs-derived cardiomyocytes expressing the GFP-ABCG2-R482G protein, than that in the parental hESC-derived cardiomyocytes. Moreover, the cell killing effect of DOX was also much less pronounced in the GFP-ABCG2-R482G expressing cells. In a compilation of three different DOX-treatment experiments, we found that at relatively low doses (3 μM), after 16 hours of DOX treatment, the parental HUES-derived cardiomyocytes had a live/dead cell ratio of 1.59 (live cells: 61,32% ± 41,11%; dead cells: 38,68% ± 36,77%), while this ratio in the HUES9-GFP-ABCG2-R482G-derived cardiomyocytes was 4.05 (live cells: 80,19% ± 13,85%; dead cells: 19,80% ± 11,30%).

## Discussion

The ABCG2 membrane protein, with divergent physiological and pathological functions, has attracted much attention in recent cell biology studies. This ABC transporter was first recognized as a multidrug efflux pump, protecting cancer cells against a wide range of chemotherapeutic agents [27], but consequent investigations documented its important role in the protection of the human body against a wide range of xeno- and endobiotics. ABCG2 is a key efflux transporter in the intestine, in the blood-brain barrier and in the placenta, and has a major role as a transporter in the liver and the kidney as well [28–30]. Regarding endobiotics, ABCG2 was shown to transport harmful porphyrin metabolites out of the human cells, and being involved in the excretion of bile acids and other toxic steroid metabolites [4,5], as well as the elimination of the end-product of human purine metabolism, uric acid. This latter role was emphasized by the GWAS studies showing the role of polymorphic variants of ABCG2 in gout formation [31,32]. Interestingly, based on mouse knock-out studies, and the presence of human individuals with no functional ABCG2 protein expression (with Jn negative blood group–see refs [33,34]), the lack of this protein does not cause a direct life-threatening effect, and only becomes important in responding to unfavorable environmental conditions, e.g. toxin exposures.

There are numerous studies regarding the role of ABCG2 in tissue-derived and pluripotent human stem cells. Hematopoietic and other regenerative tissues contain a so called side population (SP), and these were shown to be multipotent stem cells, with high repopulation capacity [35–38]. The stem cell like SP cells, found both in normal and cancerous tissues, are characterized by an increased Hoechst dye extrusion, due to a high expression of the ABCG2 transporter. It has been widely accepted that human pluripotent stem cells also express the ABCG2 transporter. Most studies (10 out of 11) showed ABCG2 expression at the mRNA level (except for Zheng H et al. [39]). It should however be noted that the protein expression levels always exhibited heterogeneous distribution, and its extent varied in examined cell lines, depending also on the culture conditions ([10,40,41]as reviewed in [9]).

Still, there are no relevant studies as yet regarding the effects of stable ABCG2 overexpression in human pluripotent stem cells and in their differentiated derivatives. Therefore in the present experiments we have decided to investigate these potential effects by a stable expression of the GFP tagged human ABCG2 protein in the human embryonic stem cell line, HUES9. We have shown earlier that the GFP-tagged ABCG2 is fully functional [12], and thus can be used both for functional and cellular localization studies. The low transfection efficiency usually characteristic for the human pluripotent stem cells, the difficulty of live cell selection and single-cell cloning, as well as the long-term cell differentiation protocols requiring special factors and conditions, were major hurdles in these experiments. Therefore we used molecular biology constructs, gene delivery and integration methods, and fluorescent GFP-tagged ABCG2 membrane proteins, which proved already usable in our earlier studies, and thus provided a supporting background for the current studies. The Sleeping Beauty transposon system used here was shown to provide preferentially random transgene integration [42], and we selected transgenic cells with low integrated copy numbers, to avoid major genetic alterations. The use of multiple transgenic hES cell clones in this study ensured that some untoward gene alteration could not alter the overall results.

As documented in detail, we found that the stable overexpression of the GFP-tagged wild-type and a mutant variant of the ABCG2 did not change the basic characteristics of hESCs. We found no differences between the parental and the GFP-ABCG2 expressing hESCs in their spontaneous and targeted differentiation capacity. However, the functional presence of the overexpressed ABCG2 protein was clearly reflected in an increased DCV dye extrusion capacity and a protection against toxic drugs, e.g. mitoxantrone and doxorubicin.

As shown here, the overexpression of a functional GFP-ABCG2 protected the hESCs against mitoxantrone toxicity (see Fig 4B). This was true both for the wild-type ABCG2 and the substrate mutant functional variant of ABCG2-R482G. Thus, by a stable expression of a relatively small amount of functional ABCG2 membrane protein, this technology allows the generation of selected toxin-resistant pluripotent human stem cells.

An important finding in our current study is the strong protective effect of ABCG2 expression in the hESC-derived cardiomyocytes against doxorubicin (DOX) toxicity (see Fig 5). Doxorubicin has a widely observed cardiotoxicity which limits its use as a potent anticancer agent. In the case of human pluripotent stem cell-derived cardiomyocytes it has been shown that DOX exposure causes dose-dependent increases in apoptotic and necrotic cell death, reactive oxygen species production, mitochondrial dysfunction and increased intracellular calcium concentrations [43]. Here we demonstrate that DOX toxicity is strongly reduced in cardiomyocytes expressing the ABCG2 protein, especially when the ABCG2-R482G variant is used, which has better DOX transporting properties than the wild-type ABCG2. Still, cardiac differentiation or contractile and signal generation functions were found to be unaltered in these cells. Since in normal human cardiomyocytes the expression of the ABCG2 transporter is practically absent [44,45], our current findings may help to devise gene-modified cardiac cells protected against toxic agents.

As a summary, we have shown here that a stable overexpression of GFP-tagged ABCG2 multidrug transporter does not change the pluripotency or the spontaneous and targeted differentiation features of human pluripotent stem cells. The use of these transgenic cells may help us to better understand the function and localization of ABCG2 during tissue differentiation from human stem cells, as well as to engineer toxin-resistant human stem cell derived tissues.

## Conclusions

We have generated human embryonic stem cells stably overexpressing the wild-type and a substrate-mutant GFP-ABCG2 fusion protein. ABCG2 overexpression did not change the pluripotency, growth, and the spontaneous or targeted differentiation properties of the hESCs. Still, the overexpression of a functional GFP-ABCG2 provided increased resistance in hESCs against mitoxantrone, and protected the hESC-derived cardiomyocytes against doxorubicin toxicity. These studies indicate that while ABCG2 expression does not significantly influence basic stem cell properties, the expression of this multidrug transporter provides toxin-resistance to pluripotent stem cells or stem cell derived tissues.

## Supporting information

S1 Fig The Sleeping Beauty (SB) transposon vector used for the stable expression of the GFP-ABCG2 fusion protein. The transposon cassette also contains a puromycin resistance gene expression unit which allowed us to select the integrated transposon containing cells by applying this antibiotic. IRDR-(L)/(R)–left and right inverted repeat direct repeat SB transposon sequences; Puro–puromycin resistance gene; Amp res–ampicillin resistance gene; F1 ori / ColE-or: bacterial replication origins; polyA: polyadenylation sequences.
(TIF)

S2 Fig mRNA expression during spontaneous differentiation of parental HUES9 cells and HUES9 cells stably expressing the GFP-ABCG2 variants. Spontaneous differentiation was performed via embryoid body (EB) formation system. After 6 days EBs were placed onto gelatin coated 24 well plates, where they underwent spontaneous differentiation. We collected samples for mRNA expression analysis before differentiation (at day 0) and at 6, 12, and 18 days of differentiation (for details see Methods). S2 Fig shows the mRNA levels of the Oct-4 and Nanog (pluripotent), AFP (endoderm), T (Brachyury) (mesoderm) and Pax6 (ectoderm) markers. The PRLP0 ribosomal protein mRNA expression was used as the internal control for quantification. Figures shows the relative mRNA levels to PRLP0 and were normalized to the undifferentiated HUES9 (d0) samples. Values represent the means±S.D. of 3 independent experiments.
(TIF)

S3 Fig mRNA expression in undifferentiated state of parental HUES9 cells and HUES9 cells stably expressing the GFP-ABCG2 variants. We collected samples for mRNA expression analysis before differentiation and measured the expression levels of the ABCG2, ABCB1 and ABCC1 transporters. The PRLP0 ribosomal protein mRNA expression was used as the internal control for quantification. Values represent the means±S.D. of 2 independent experiments.
(TIF)

S4 Fig mRNA expression in undifferentiated state and after a directed hepatocyte differentiation of parental HUES9 cells and HUES9 cells stably expressing the GFP-ABCG2 variants. We collected samples for mRNA expression analysis before differentiation (“stem” samples) and at 18 days of differentiation (“hepatic” samples) (for details see Methods). We measured the expression levels of the Oct-4, AFP, ALB, ABCB11 and HNF4 markers. The PRLP0 ribosomal protein mRNA expression was used as the internal control for quantification. Values represent the means±S.D. of 2 independent experiments.
(TIF)

S5 Fig Directed differentiation of HUES9 cells expressing GFP-ABCG2 into hepatocytes. Immunostaining analysis of CK18 and HNF4 hepatocyte markers by confocal microscopy. Co-immunostaining of CK18 or HNF4 and GFP-ABCG2 in hepatocytes differentiated from HUES9 cells. Anti-GFP: green, CK18 or HNF4: red, nuclei: blue.
(TIF)

S1 Table Mitoxantrone cytotoxicity in EGFP-HUES9 (control) cells and in HUES9 cells expressing GFP-ABCG2 variants. The ratio of the dead and living cells was calculated on the basis of propidium-iodide accumulation and was normalized to untreated cells. Values represent the means±S.D. of 3 independent experiments. Significant differences (Student’s t-test, P<0.01) in the survival of parental and ABCG2-variants expressing clones are indicated by asterisks.
(TIF)

S1 Video HUES9-GFPG2-R482G beating cardiomyocytes. (MP4)

S2 Video HUES9 beating cardiomyocytes. (MP4)
