# Supplementary figures and images for: Generation of multidrug resistant human tissues by overexpression of the ABCG2 multidrug transporter in embryonic stem cells
Source: PLoS One. 2018 Apr 12;13(4):e0194925. doi: 10.1371/journal.pone.0194925 (PMC5896897; doi:10.1371/journal.pone.0194925)

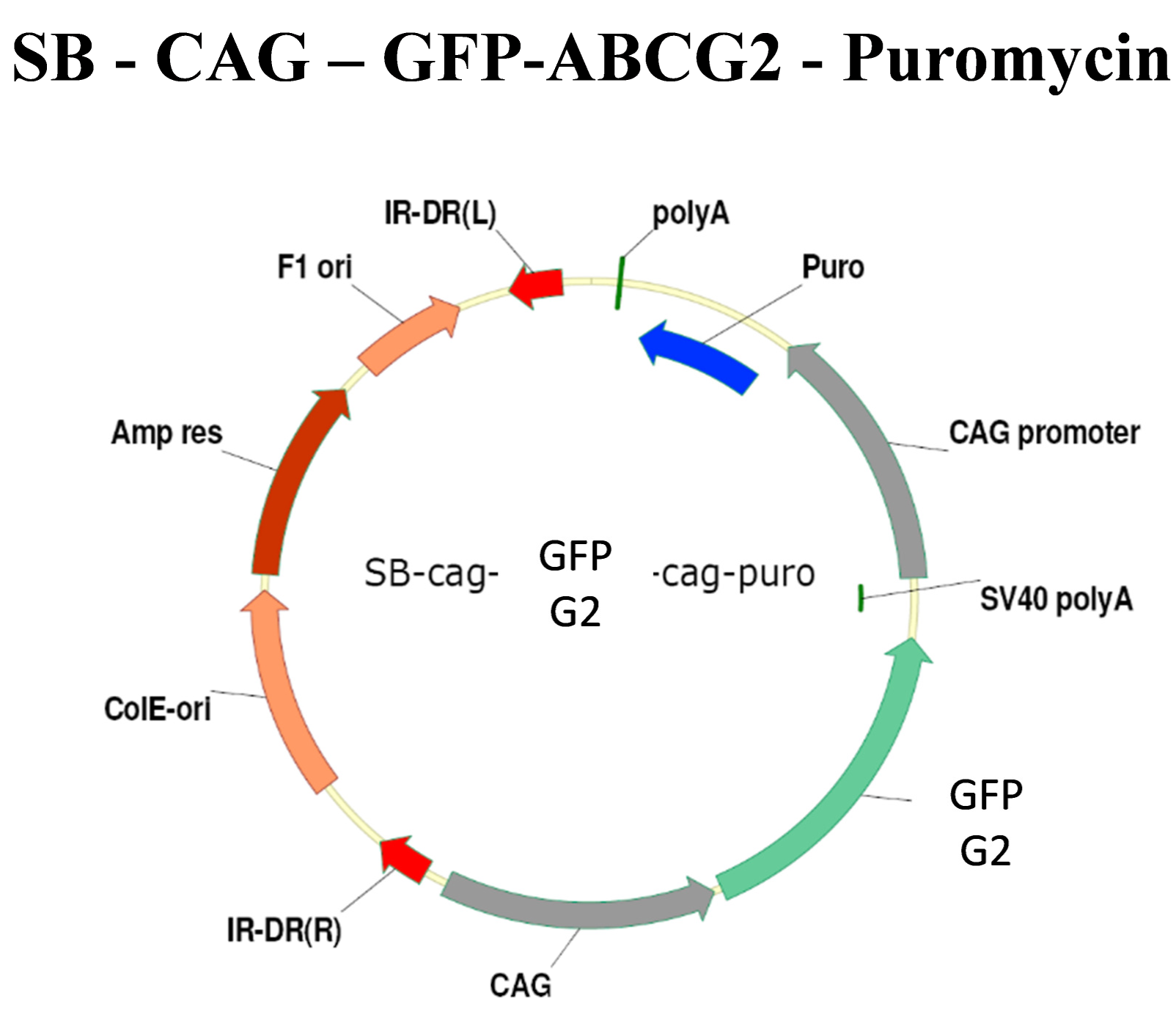

Supplement: S1 Fig — The transposon cassette also contains a puromycin resistance gene expression unit which allowed us to select the integrated transposon containing cells by applying this antibiotic. IRDR-(L)/(R)–left and right inverted repeat direct repeat SB transposon sequences; Puro–puromycin resistance gene; Amp res–ampicillin resistance gene; F1 ori / ColE-or: bacterial replication origins; polyA: polyadenylation sequences. (TIF) [file pone.0194925.s001.tif]

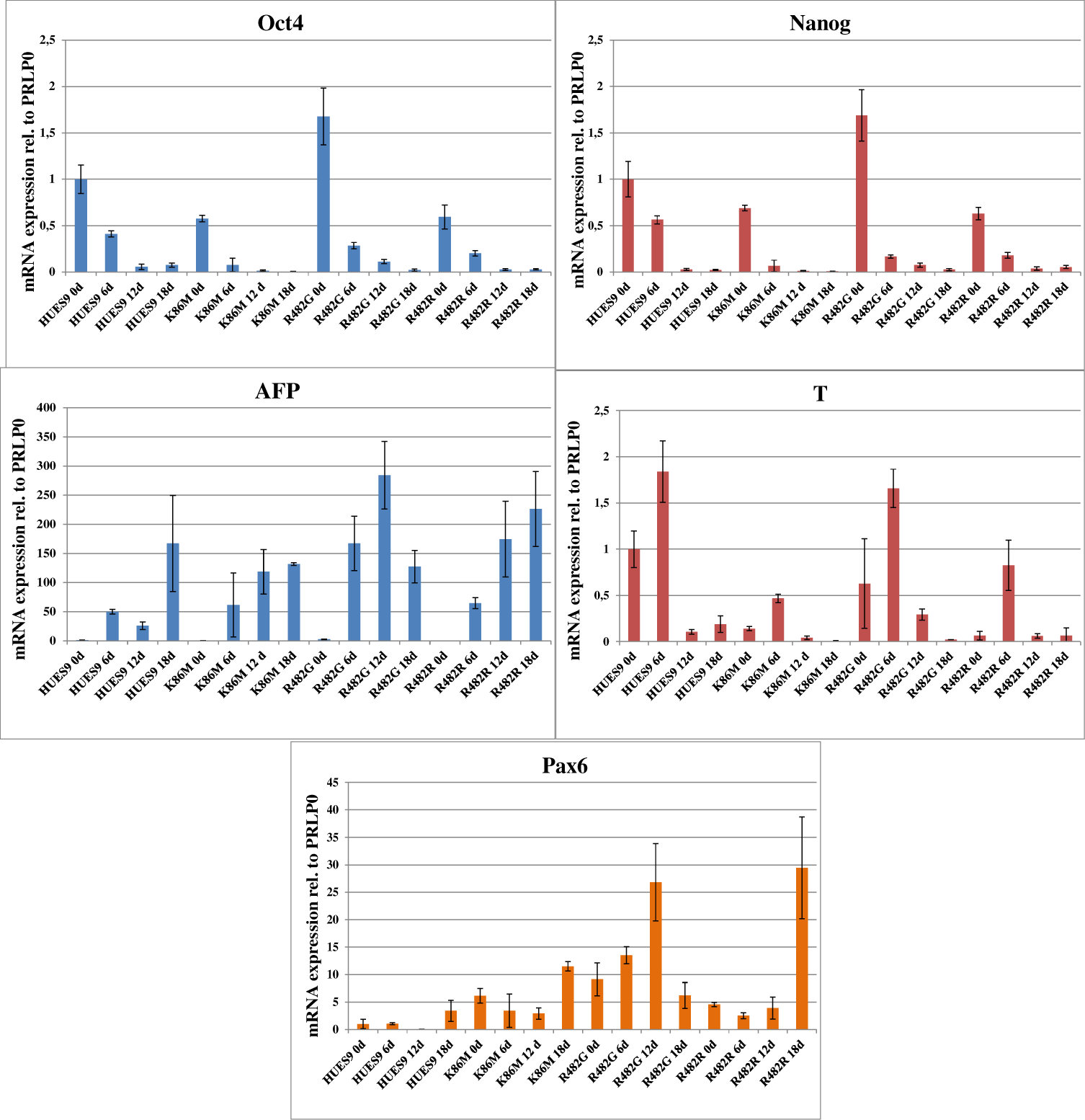

Supplement: S2 Fig — Spontaneous differentiation was performed via embryoid body (EB) formation system. After 6 days EBs were placed onto gelatin coated 24 well plates, where they underwent spontaneous differentiation. We collected samples for mRNA expression analysis before differentiation (at day 0) and at 6, 12, and 18 days of differentiation (for details see Methods). S2 Fig shows the mRNA levels of the Oct-4 and Nanog (pluripotent), AFP (endoderm), T (Brachyury) (mesoderm) and Pax6 (ectoderm) markers. The PRLP0 ribosomal protein mRNA expression was used as the internal control for quantification. Figures shows the relative mRNA levels to PRLP0 and were normalized to the undifferentiated HUES9 (d0) samples. Values represent the means±S.D. of 3 independent experiments. (TIF) [file pone.0194925.s002.tif]

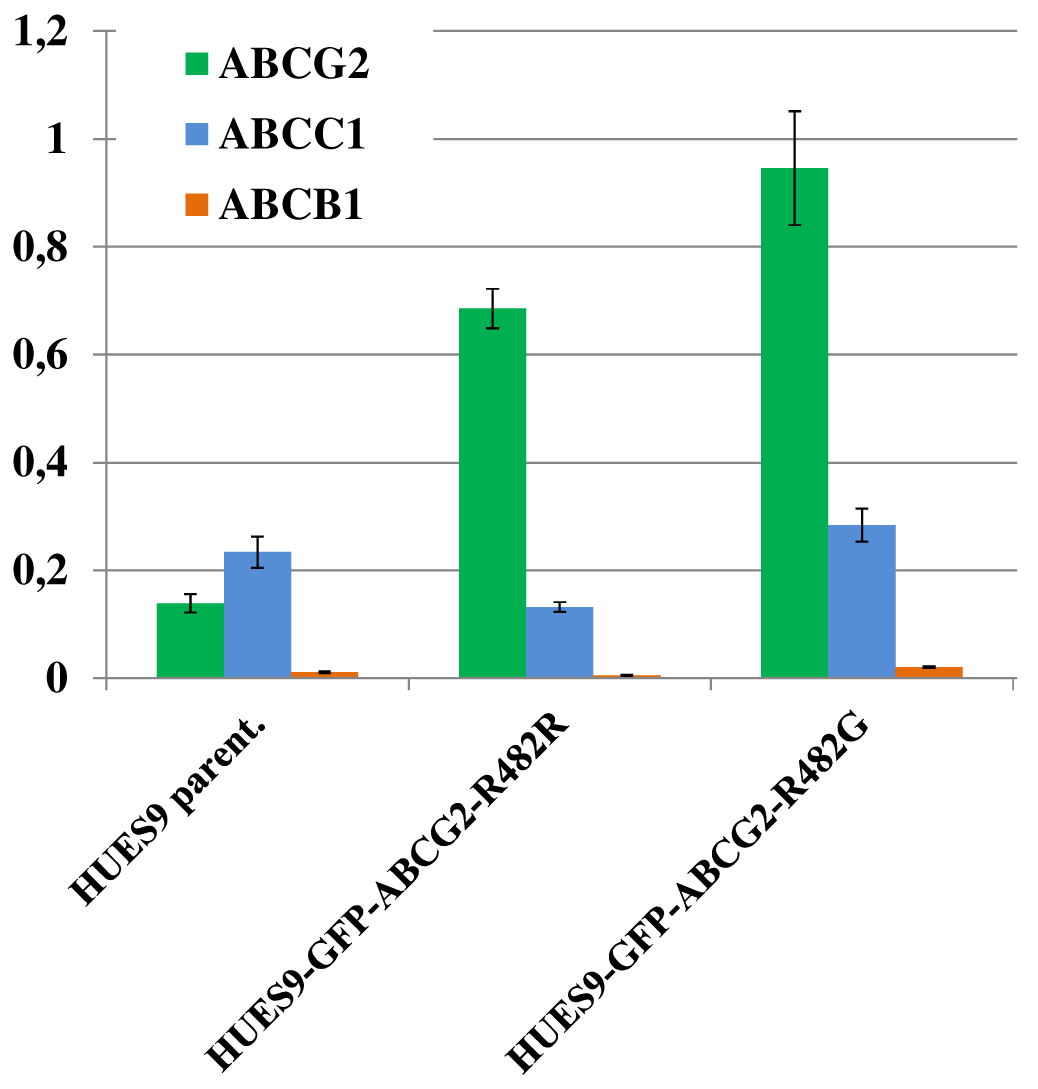

Supplement: S3 Fig — We collected samples for mRNA expression analysis before differentiation and measured the expression levels of the ABCG2, ABCB1 and ABCC1 transporters. The PRLP0 ribosomal protein mRNA expression was used as the internal control for quantification. Values represent the means±S.D. of 2 independent experiments. (TIF) [file pone.0194925.s003.tif]

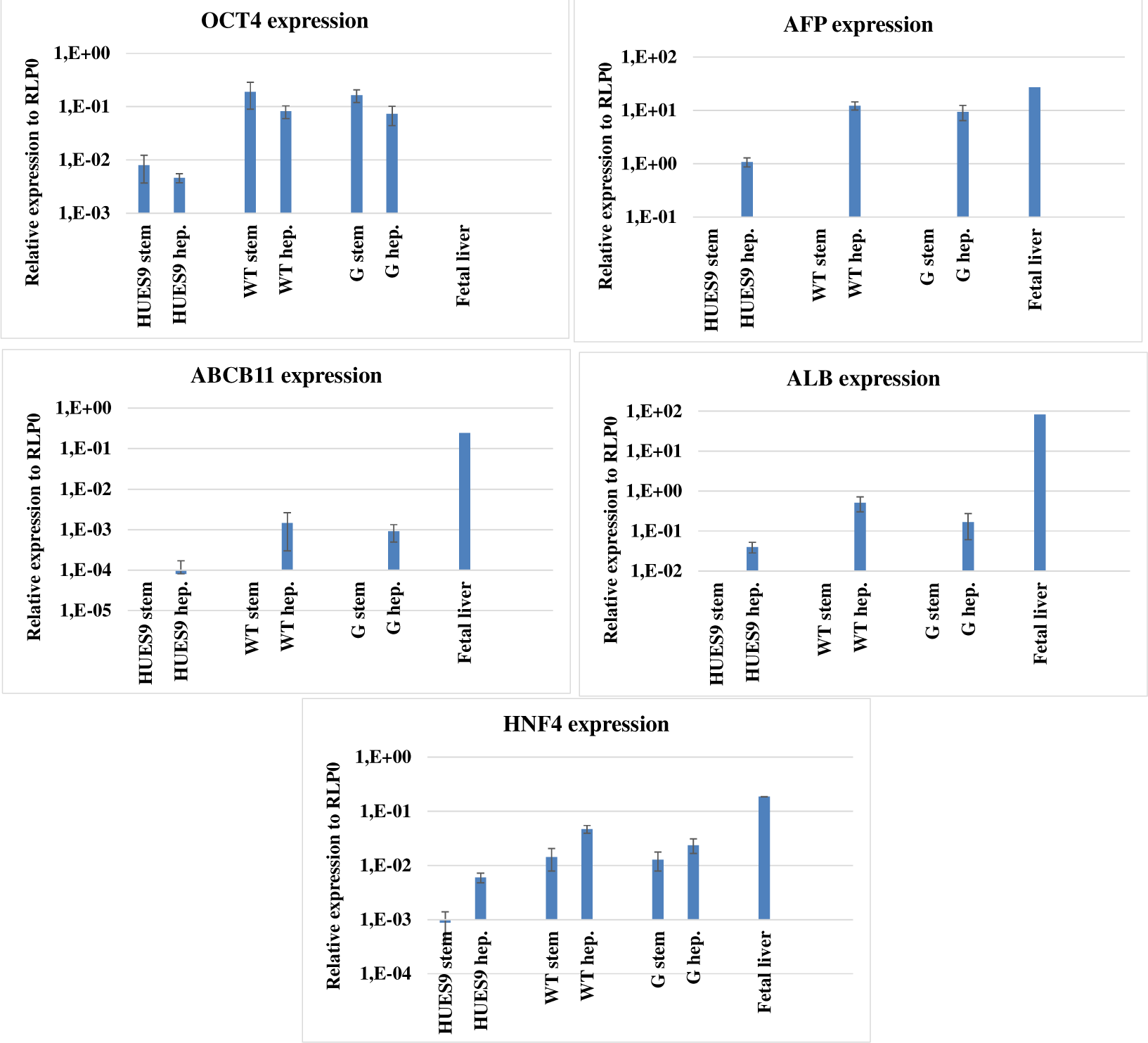

Supplement: S4 Fig — We collected samples for mRNA expression analysis before differentiation (“stem” samples) and at 18 days of differentiation (“hepatic” samples) (for details see Methods). We measured the expression levels of the Oct-4, AFP, ALB, ABCB11 and HNF4 markers. The PRLP0 ribosomal protein mRNA expression was used as the internal control for quantification. Values represent the means±S.D. of 2 independent experiments. (TIF) [file pone.0194925.s004.tif]

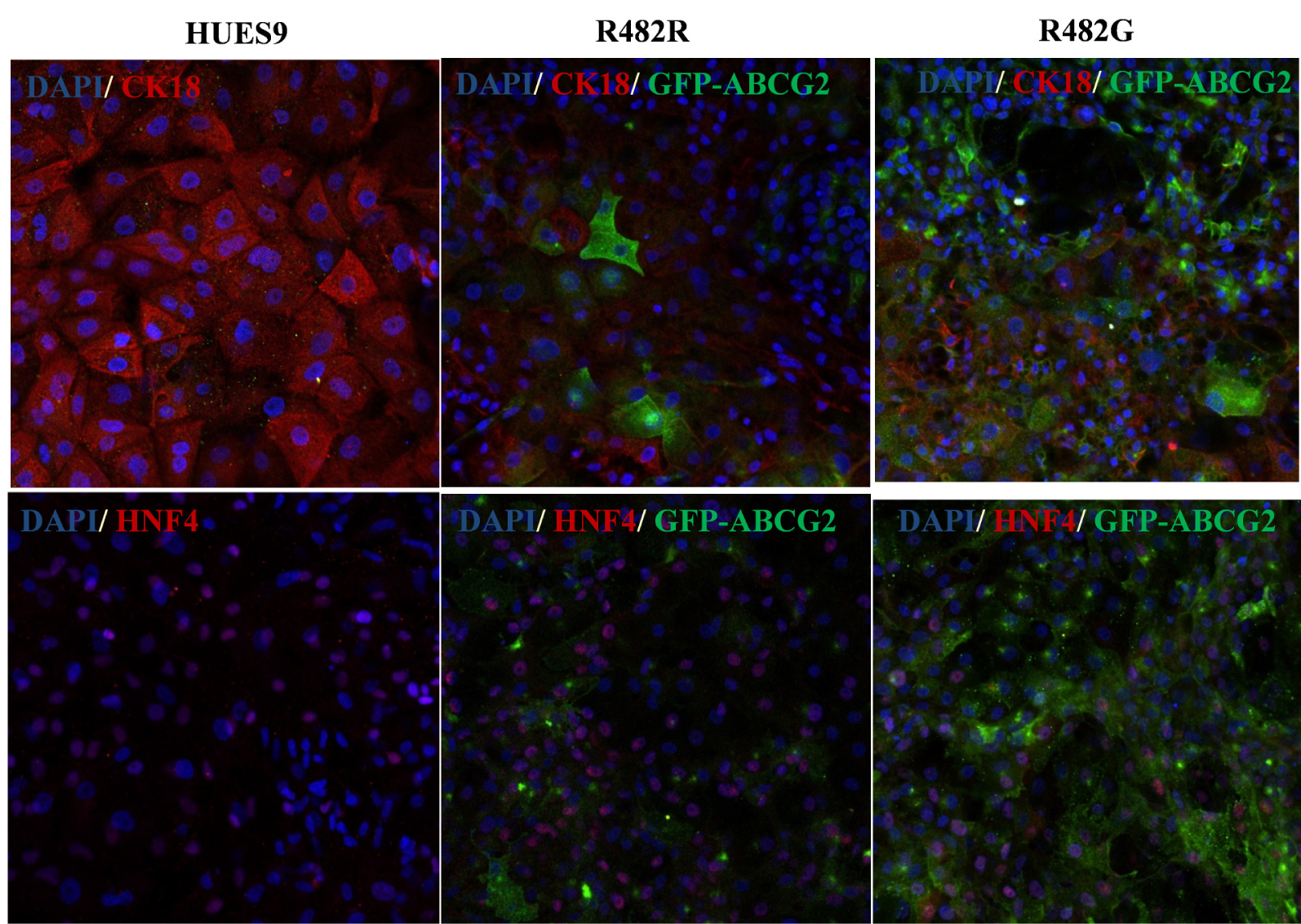

Supplement: S5 Fig — Immunostaining analysis of CK18 and HNF4 hepatocyte markers by confocal microscopy. Co-immunostaining of CK18 or HNF4 and GFP-ABCG2 in hepatocytes differentiated from HUES9 cells. Anti-GFP: green, CK18 or HNF4: red, nuclei: blue. (TIF) [file pone.0194925.s005.tif]

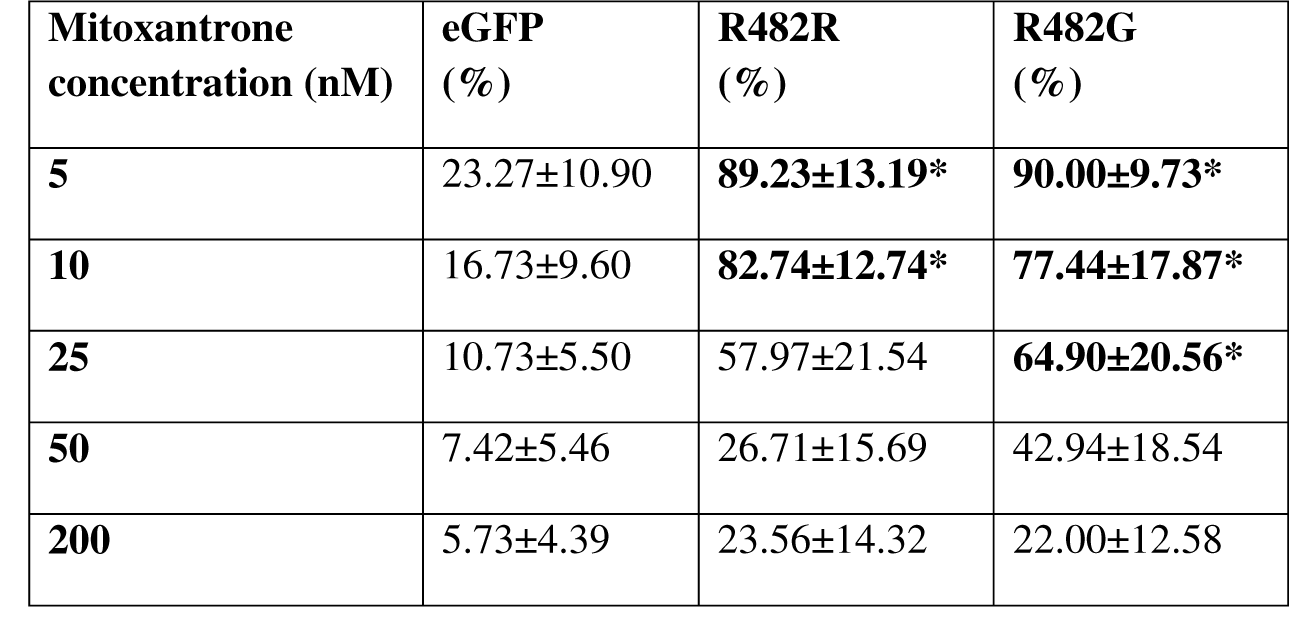

Supplement: S1 Table — The ratio of the dead and living cells was calculated on the basis of propidium-iodide accumulation and was normalized to untreated cells. Values represent the means±S.D. of 3 independent experiments. Significant differences (Student’s t-test, P<0.01) in the survival of parental and ABCG2-variants expressing clones are indicated by asterisks. (TIF) [file pone.0194925.s006.tif]
